# Using gloves wisely: rethinking glove use for health, safety, and environmental sustainability

**DOI:** 10.1017/ash.2026.10323

**Published:** 2026-04-01

**Authors:** M. Lauren Lalakea, Radhika Sheth, Elizabeth Cerceo, Yvonne Huckleberry, Preeti Jaggi, Vidya Mony, Elizabeth Monsees

**Affiliations:** 1 Department of Otolaryngology- Head and Neck Surgery, Stanford University, USA; 2 Department of Infectious Diseases, Henry Ford Health System, USA; 3 Department of Medicine, Cooper Medical School of Rowan University, USA; 4 Department of Pharmacy, Banner University Medical Center-Tuscon, USA; 5 Department of Pediatrics, Division of Infectious Diseases and Children’s Healthcare of Atlanta, Emory University, USA; 6 Department of Pediatrics, Division of Infectious Diseases, Santa Clara Valley Healthcare, USA; 7 Patient Care Services Research, Children’s Mercy, University of Missouri Kansas City School of Medicine, USA

## Abstract

Non-sterile gloves (NSG) are essential for reducing transmission of pathogens and for protecting patients and staff. Their overconsumption can lead not only to an increased risk of cross-contamination but also increased plastic waste and downstream environmental effects. Glove reduction campaigns worldwide have safely and successfully reduced their inappropriate use and associated waste. In this multidisciplinary commentary, we discuss the sweeping public health and environmental consequences of NSG overuse and strategies to implement a successful glove stewardship campaign.

## Introduction

The United States (US) healthcare sector is responsible for 8.5% of the nation’s total greenhouse gas emissions (GHGe) annually, contributing to climate change and resulting in an estimated 388,000 disability-adjusted life years lost in 2018.^1^ Supply chain goods and services account for approximately 82% of these emissions, and single-use devices comprise a large portion of that category.^1,2^ Non-sterile gloves (NSG) are among the highest volume single-use plastic products purchased by health systems. The COVID-19 pandemic caused a surge in use, with utilization expected to double over the next five years.^3,4^ Many health professionals trained during this period of heightened personal protective equipment (PPE) use, resulting in a generation of clinicians accustomed to wearing gloves by default. While an indispensable component of PPE, NSG overconsumption significantly exacerbates the carbon footprint of PPE.^5,6^

The World Health Organization World Hand Hygiene Day 2025 highlighted the appropriate use of gloves and the environmental and climate impacts stemming from their overuse.^7^ Despite clear guidelines, inappropriate glove use is common among healthcare workers (HCW).^8,9^ A recent review examined gloves’ role in infection prevention (IP) as essential PPE, but also as a possible impediment to effective practice, emphasizing that their overuse can reduce hand hygiene (HH) compliance and increase the risk of healthcare-associated infections (HAI).^8–10^ The imperative to improve healthcare sustainability has led to growing interest in reducing inappropriate glove use while maintaining staff and patient safety.^11–15^

In this commentary from a multidisciplinary group of authors, we examine the consequences of inappropriate NSG use, review glove reduction campaigns, and provide practical strategies for implementing their safe and effective use in support of personal, patient, public, and planetary health.

## Impact of glove overconsumption

### Effects on infection prevention

Gloves serve as a primary barrier to protect HCWs’ hands from gross contamination with body fluids and to minimize the risk of transmission of pathogens between patients and environments. While important for contact precautions, this commentary focuses on NSG use in the context of standard precautions. Their overconsumption in specific transmission-based precautions also needs reassessment but is beyond the scope of this study. Under standard precautions, glove use is indicated for anticipated contact with body fluids, mucosa, open wounds or skin breaks, and hazardous materials (including sharps, contaminated devices, certain drugs and caustic cleaning materials).^16–18^

Notably, HCWs use gloves inappropriately in up to 50% of clinical encounters, e.g., they are worn when not indicated, for a longer time than needed, or not changed when required between tasks, leading to potential cross-contamination.^19–22^ The efficacy of gloves in IP relies on their proper use, and this advantage is lost if HH is neglected before donning, if gloves are ill-fitting, or if hands are contaminated during removal. The revised Society for Healthcare Epidemiology in America (SHEA) compendium provides guidance on glove use and HH.^23^

Open glove boxes can be a source of contamination, as pathogenic bacteria from the hands may be introduced during glove retrieval.^24^ Even gloves from unopened boxes have been shown to have bacterial loads comparable to those on hands following HH.^25,26^ Moreover, glove usage has been associated with reduced HH compliance.^8,27,28^ HH is the cornerstone of HAI prevention and gloves are not a substitute for HH. Girou et al. demonstrated that 59% of gloves sampled after patient interactions were contaminated with the same pathogenic bacteria colonizing the patient, indicating a risk of cross-contamination, and highlighting that gloves are not an infallible barrier against bacteria.^9^

Additionally, prolonged glove use has been associated with occupational health risks, including dermatitis which has been reported by up to 25% of HCWs.^29^ Although nitrile gloves, now almost half of the market, are less allergenic than latex, they can still cause irritation and allergic contact dermatitis.^30–32^

When used inappropriately, gloves may become occupational hazards or vectors of transmission that may undermine HH and IP efforts.

### Environmental, financial, and social considerations

In the US alone, an estimated 100–124 billion examination gloves are used annually, representing 350,000–434,000 tons of medical plastic waste.^12^ Gloves are sometimes discarded as regular landfill waste or treated as biohazardous material, requiring energy-intensive autoclaving prior to landfill, or incineration, the latter producing harmful pollutants.^33^ In landfills, gloves contribute to microplastic contamination, and leach endocrine-disrupting chemicals, heavy metals, and other toxic substances into the environment, with downstream ecological and health impacts.^34–37^ NSGs account for approximately 45% of the carbon footprint of PPE, with GHGe estimated at 26–34 gm of carbon dioxide equivalent (CO2e) per individual glove.^5,38^ Based on current consumption, glove-related GHGe in the US total roughly 2.6–4.2 million metric tons of CO2e annually, equivalent to 6.65–10.6 billion miles driven in an average gas-powered car.^39^ Thus, curbing unnecessary glove use is both a public and a planetary health imperative.

In North America, the medical glove market was valued at $2 billion in 2024.^40^ NSG costs in the US are in the range of $0.05 per glove. For a California healthcare system comprising of three hospitals and fifteen clinics, costs totaled $2.2 million for 41 million nitrile gloves purchased in 2023.^12^ Given the ubiquity of NSGs, even modest reduction in their overuse has the potential for significant financial savings.

The social costs associated with glove overuse include human rights implications. The production and supply chain for NSGs is rife with concerns about labor rights abuses and exploitation. Many NSGs are sourced from Malaysia, where migrant workers in glove manufacturing have been subject to debt bondage, retention of identity documents, squalid working conditions, and excessive working hours.^41,42^ These conditions have previously led to a temporary ban in the US on imports from several glove manufacturers.

A second social “cost” is the possible negative impact gloved hands can have on human touch and connection. An observational study reported that glove use can sometimes be driven by HCWs perception that the patient is “dirty” and this may lead to an implicit stigma.^20^ As one physician reflecting on his own methicillin-resistant *Staphylococcus aureus* infection observed, gloves function can be a psychological barrier that “creates a small distance between the doctors and their patients.”^43^ Social touch may express empathy, compassion, and establish trust in clinical encounters, and it can enhance the patient-clinician relationship.^44–48^ Moreover, there has been increasing evidence supporting the therapeutic value of touch across many disciplines and age groups.^49,50^

### Review of glove use reduction campaigns

In response to increasing concerns regarding potential adverse impacts of NSG overuse, there is an emerging body of evidence supporting the effectiveness and safety of glove reduction campaigns (Table 1).^7,10,41,51^ The first of these was “The Gloves are Off” campaign in a pediatric hospital in the United Kingdom (UK) in 2018.^14,52^ This facility-wide inpatient campaign reported an impressive reduction in glove use (33%), waste, and cost, with no increase in HAIs. This success was replicated by another pediatric hospital in the UK that further noted an annual savings of 286 metric tons of CO2e.^15^ Two additional initiatives within specific inpatient units in Canada and Australia have documented similar results: both noted improvements in appropriate glove use, and one demonstrated an increase in HH compliance.^11,53^ Two studies have reported favorable results in outpatient settings.^12,13^ Informal staff feedback included reports of improved attention to HH, the value of touch in communicating care and acceptance, and positive feelings and sense of agency engendered by working to reduce plastic waste and environmental harms associated with medical care delivery.^12^

**Table 1.** Glove reduction campaigns

Summary of glove use reduction literature								
Campaign/ authors (year)	Country	Setting	Summary of interventions	Glove use outcome measures	Cost reduction	Waste reduction	Emissions reduction	Other
**“Gloves are off!”, Leonard et al. (2018, 2019) ^and 14,52^**	UK	Pediatric hospital	Education booth in cafeteria, educational material including FAQ, webpages, screensavers, posters	3.7 million fewer gloves ordered/yr (33.3% reduction)	£90,000 GBP/yr	18 metric tons/yr	–	No increase in HAIs; fewer staff visits for hand/skin problems
**“Gloves off”, Durham et al. (2022)^15^**	UK	Pediatric hospital	Engagement activities with patients and staff, including campaign posters	11 million fewer gloves/yr (14.6% reduction)	£14,000 GBP/yr	40 metric tons/yr	286 tons/yr	Educational activities targeted patients, focus on negative effects of plastic pollution on sea and aquatic life
**BC GreenCare (2024)^53^**	Canada	Cardiac ICU	Educational material; audit tool; peer-to-peer education	90,100 gloves/6 mo (53% reduction)	–	–	2,342.6kg/ 6 mo	Appropriate glove use compliance increased from 39% to 75%
**“Gloves Off!”, Wilkie-Miskin et al. (2025)^11^**	Australia	Inpatient surgical wards	Visual reminders with signs, posters, badges; “music video”; face-to-face education sessions	6.9 glove/patient- day fewer gloves purchased; 13,020 fewer gloves/mo (37% reduction)	AUD$651 /mo	44.8 kg/mo	443 kg/mo	Improved hand hygiene compliance from 59% to 83%; improvement in staff knowledge regarding appropriate glove use on postsurvey
**Smith et al. (2025)^13^**	UK	Sexual Health and HIV clinics	Presurvey; targeted staff education; posters; videos; patient-facing posters	78,310 fewer gloves/yr (45.2% reduction in purchasing)	£1200 GBP/yr	–	–	62.5% of staff reported decrease in personal glove use postintervention
**“Use Gloves Wisely”, Lalakea et al. (2025)^12^**	US	Outpatient ENT and plastic surgery/ burn clinics	Visual reminder with signs and posters; education at meetings and nursing huddles	56,628 fewer gloves/yr (27% decrease in glove use/visit)	$3003.17/yr	180.2 kg/yr	1,472–1767 kg/yr	Staff highlight better hand hygiene habits, value of touch, and environmental benefit

AUD, Australian Dollar; ENT, Ear, nose, and throat; GBP, Great Britain pound; HAI, Healthcare-associated infections; HIV, Human Immunodeficiency Virus; Kg, kilogram; Mo, month; UK, United Kingdom; US, United States; yr, year.

Although studies differed in methods and the degree of reduction achieved, findings were consistently positive for waste reduction, environmental impact, and cost savings across settings. Studies reported improved glove use and HH compliance, enthusiasm for reducing environmental impacts, improved staff knowledge, and an anecdotal endorsement for enhanced human connection through touch. These findings reinforce that glove reduction is feasible and beneficial across multiple domains of patient safety, healthcare finance, and environmental stewardship.

### Strategies for operationalizing glove reduction campaigns

Motivating behavioral change for NSG overuse requires a thoughtful approach (Table 2). Assessing and addressing barriers are key initial steps. Overuse may be driven by habit, culture, emotions, workflow pressures, or misconceptions about safety.^20^ A recent study of human factors in the misuse of NSGs found that the HCW’s decision to wear gloves was strongly influenced by emotions of fear and disgust and a need for self-protection. There was also a strong sense among HCWs that wearing NSG is a personal decision that others have no authority to influence.^54^ In previous glove reduction initiatives, perceptions of patient uncleanliness and personal and patient safety were cited as primary staff concerns.^12,13^ Moreover, “gloves off” was perceived as a threat to professional autonomy, while the positive framing of “Use Gloves Wisely” as an invitation for resource stewardship was more readily accepted.^12^ While emotional responses and culture may be difficult to change, staff knowledge gap about safety and glove use are more amenable to targeted educational intervention.^11,13^ Employees should be assured that the facility’s HH and glove-use policies are grounded in evidence-based practices and are aligned with established IP guidelines.

**Table 2.** Strategies to operationalize glove reduction campaigns

Operationalizing use gloves wisely campaigns	Possible actions, examples
**1. Identify campaign champions**	Front line HCW (including nursing, physicians, APPs, pharmacists), and environmental services Infection Prevention and hospital epidemiology Executive sponsorship is essential
**2. Gather baseline data**	Glove use or purchase data, facilitywide or individual units Estimate quantity of waste and/or emissions Annual financial costs Baseline HH and HAI rates Review internal HH/glove policies
**3. Consider barriers**	E.g., safety concerns, knowledge gaps, habit
**4. Outline key benefits/motivations, the “Why”**	Improve patient safety Improve hand hygiene Reduce risk of contamination Reduce harmful plastic waste Reduce the carbon footprint of medical care, reducing GHGe Enhance compassion/connection via touch Reduce cost
**5. Identify launch site(s)**	Consider initial launch on unit(s) with high hand hygiene rates, engaged staff
**6. Engage stakeholders**	Initial pitch presentation, including information from 2. & 3. HH/glove use guidelines Identify and address questions, concerns, knowledge gaps Identify local champions
**7. Set goals, possible outcome metrics**	Percentage glove reduction overall, or by glove/patient day, glove/clinic visit HH compliance +/− inappropriate glove use +/− HAI rates +/− Staff knowledge, use patterns, feedback Cost savings Waste avoided +/− reduced GHGe
**8. Develop targeted site-specific guidelines with local stakeholders**	What can unit members clearly designate as gloves ‘off’ and gloves ‘on’ scenarios Allow for HCW judgment in gray areas based on experience and situational factors
**9. Develop and share educational materials**	Posters Videos Presentations
**10. Monitor glove use metrics, provide feedback**	Share interval data at meetings, huddles Address additional barriers, concerns Provide ongoing support
**11. Calculate impacts**	Share successes! Additional interventions to improve results, as needed

APPs, Advanced practice providers; GHGe, Greenhouse gas emissions; HAI, Healthcare-acquired infection; HCW, Health care worker; HH, Hand hygiene.

Identifying motivating factors to inspire appropriate glove use is also important. This may include possible improvements to HH compliance and personal/patient safety, reduction in plastic waste, carbon emissions, associated environmental and public health harms, and financial savings. Baseline glove use data can illustrate the magnitude of local use and downstream volume of plastic waste. Educational material should address gaps in knowledge and highlight the broader health impacts of plastic pollution and carbon emissions to promote thoughtful resource stewardship. While all initiatives listed in Table 1 had reduced environmental impact as one of their stated objectives, one highlighted the benefit of unit ‘green champions’ in their success, and two specifically commented that staff were motivated to change their glove use practices by the prospect of positive environmental outcomes.^12,13,52^ Finally, framing glove reduction as a means to restore authentic nonverbal communication can help clinicians see the practice as enhancing and not compromising patient care or safety.

Securing executive leadership buy-in is essential for long-term success. Direct cost savings from reduced glove procurement, waste disposal, and alignment with system-wide sustainability goals, along with potential for improved patient satisfaction scores and associated quality incentives support a strong business case.

**Figure 1.**
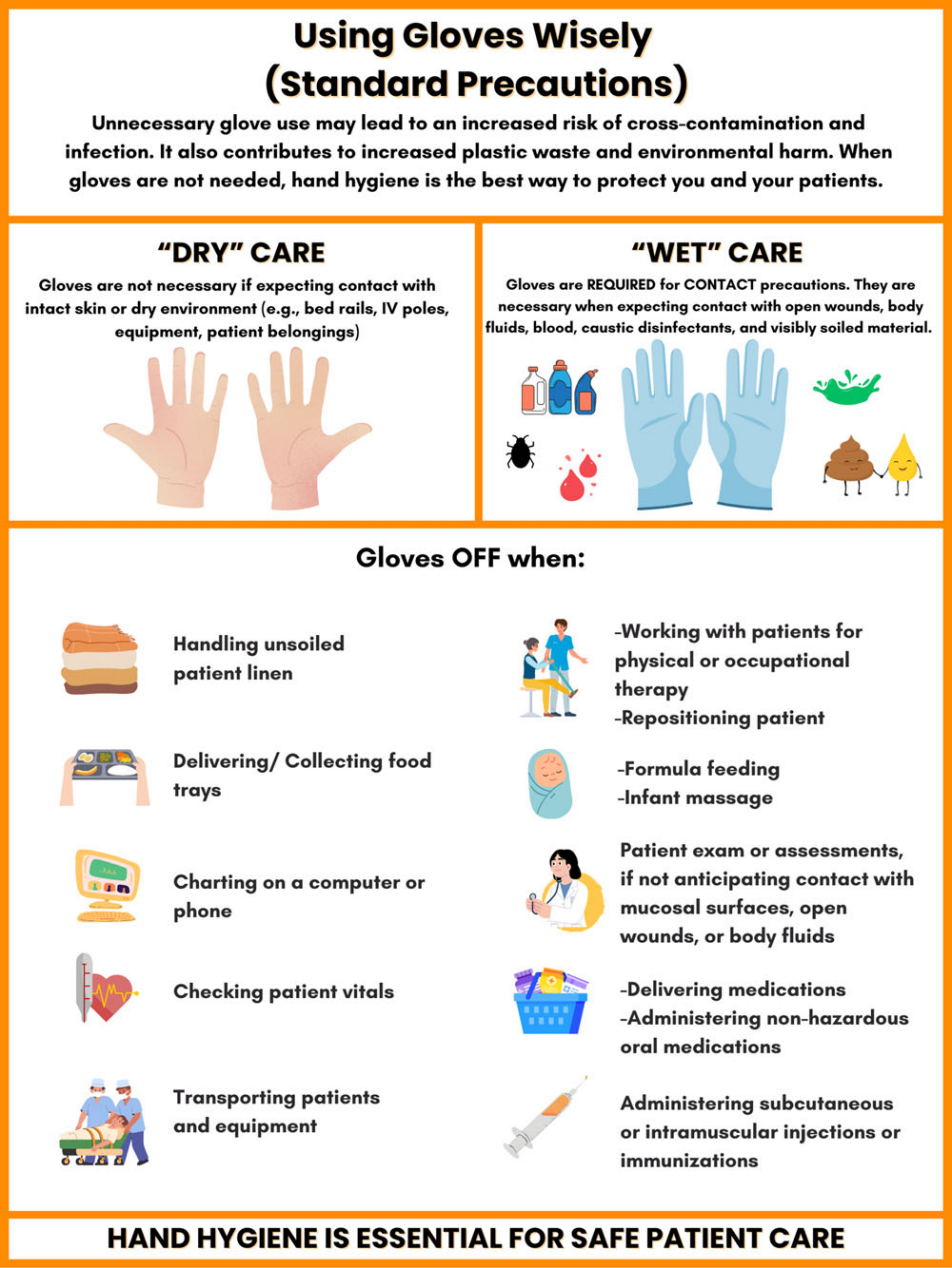
Guidance for glove use in specific clinical scenarios.

Successful campaigns rely on stakeholder engagement and developing context-specific guidelines and materials.^10^ Teams should identify and mutually agree upon clear scenarios where gloves “come off” and where they are mandatory (Figure 1). Allowing “gray” areas—where personal choice informs nuanced decisions based on situational factors—acknowledges individual professional judgment and encourages staff buy-in. Initial interventions can include staff presentations and creating simple visual aids for the unit, supported by brief educational assessments and feedback. Piloting glove reduction in a single unit may help assess feasibility and receptiveness before broader implementation. Monitoring and sharing outcomes support staff engagement. Collaborating with front-line clinicians, infection preventionists, nursing leadership, and pharmacists can facilitate practical, locally relevant guidance and foster a sense of ownership.^13^ These partnerships also reinforce a shared responsibility to ensure that the human rights consideration in glove manufacturing and procurement are explicitly recognized and upheld during purchasing decisions.

## A specialty-driven approach to reduce inappropriate glove use (Table 3)

### Direct patient contact

All staff must wear gloves in compliance with isolation precautions, e.g., contact or contact-plus precautions, and in scenarios where exposure to blood, body fluids, non-intact skin, or hazardous material (caustic disinfectants, certain medications) is anticipated. However, most components of the physical examination do not routinely require the clinician to wear gloves, e.g., taking vitals, auscultation of heart and lung sounds, neurological tests, and palpation of the abdomen. This also applies to physical and occupational therapists, who often perform manual therapy techniques, including massage therapy. Gloves may interfere with tactile sensation and palpation skills in addition to social connection.^55^ To make decisions on when to don gloves easier, we classify clinical care scenarios into “wet contact” and “dry contact” and offer clear guidance on specific scenarios (Figure 1).

**Table 3.** Specialty-driven opportunities to reduce inappropriate non-sterile glove use

Specialty/role	Unique contributions to glove stewardship
Nursing	Model behavior while following standard precautions Patient education Initiate and support culture change
Physicians	Model behavior for trainees and other team members Integrate stewardship into bedside and didactic teaching Engage with systemwide, regional, and national committees to lead and promote stewardship initiatives
Pharmacists	Align practice with CDC/OSHA guidance Medication safety leadership
Infection prevention	Develop and endorse evidence-based policies around glove stewardship Educate staff and monitor compliance Lead or support glove stewardship campaigns
Antimicrobial stewardship/infectious diseases	Integrate sustainability principles into antimicrobial stewardship Support infection prevention teams with glove stewardship campaigns Engage with local and national organizations to promote evidence-based guidelines
Professional organizations	Organize physicians and nurses to collaborate, share knowledge and best practice guidelines, and promote stewardship policies Incentivize reducing unnecessary glove use and plastic waste by offering recognition to hospitals who monitor these metrics

### Nursing

Beyond physical assessments, nurses administer vaccinations, feed and comfort patients, which do not require glove use. As central figures in care delivery, nurses can model proper adherence to standard precautions, including HH, helping to break the habit of ubiquitous gloving. Because patient education is a core responsibility, engagement in professional organizations like the American Nurses Association (ANA) and Alliance of Nurses for Healthy Environments can equip nurses to promote appropriate glove use and broader sustainability strategies.^56^

### Pharmacists

There are opportunities for pharmacists to reduce unnecessary gloving within inpatient and outpatient settings. Although the CDC recommends HH without glove use before preparing and administering vaccines, gloving for all vaccine administration has become common despite limited evidence. Occupational Safety and Health Administration guidelines note that glove use is not indicated for vaccine delivery unless the provider has broken skin on their hands.^57–59^ Pharmacists should reassess glove use during patient contact and counseling, non-hazardous medication preparation and delivery, and home health visitation, where frequent, proper HH offers equal protection.

### Physicians

Physicians are seen as the leaders of the healthcare team and trainees often mirror their attending physicians’ behavior. Glove use is often influenced by social pressure.^20^ In a survey of 226 medical trainees, 40% of respondents reported always using gloves as part of standard precautions and 55% of residents reported that they were taught to glove before all patient care encounters.^60^ Modeling evidence-based glove use can be a powerful opportunity to reshape team practices. Trainees increasingly recognize climate change as a growing concern and feel their education inadequately addresses healthcare sustainability.^61^ Physicians can integrate glove use and other sustainability topics into bedside teaching and didactic curricula. They can engage with hospital sustainability committees or local and national climate-focused professional organizations, such as the Medical Society Consortium on Climate and Health (MSCCH) and Health Care Without Harm (HCWH) which mobilize clinicians across the US and are influential in shaping sustainability standards.

### Antimicrobial stewardship teams

Antimicrobial stewardship (ASP) teams and Infectious Disease (ID) specialists can be key stakeholders in glove reduction campaigns, as both stewardship and sustainability focus on managing resources and building resilience. ID physicians play an important role in advocating for HH education and compliance, and much like the IP team, their expertise can be leveraged to ensure that glove use aligns with necessity and not simply with habit. They can also engage with national societies, like the Pediatric Infectious Disease Society (PIDS) and SHEA that have put out a call for action to the ID community to address climate change.^62,63^

### Infection prevention team

Infection Preventionists have dual responsibility to develop system-level recommendations that influence patient and staff safety and interface with clinical teams to understand barriers to practice. This bidirectional perspective strengthens IP expertise in risk detection. They can reinforce proper glove use, HH practices, strategies to mitigate hand irritation, and address other influences on gloving decisions, such as patient expectations or a desire to appear safe.^19,64^

Revised SHEA guidelines call for competency-based training to avoid self- and environmental contamination when gloves are worn, yet only 24% of clinicians at a large health system reported sufficient education on PPE’s environmental impacts.^23,65^ IP teams are instrumental in delivering this training, compiling compliance data, guiding product selection, monitoring use and disposal, and linking patient and staff safety with sustainability efforts. Given their expertise, IP endorsement is critical to glove reduction campaigns.

IP teams can support the adoption of evolving evidence-based practices and can leverage resources from societies, such as SHEA and PIDS, which promote sustainability and connect climate action to IP.

### Professional organizations

Medical societies play a key role in converting emerging evidence into clear guidance and then disseminating it to promote practice change. Specialty societies such as the Infectious Disease Society of America (IDSA), SHEA, PIDS, and ANA can define clinical scenarios where gloves are and are not indicated and reinforce HH practices. Incorporation of society recommendations into consensus guidelines can support clinician confidence, reduce ambiguity, address misconceptions driving overuse, and embed glove stewardship within IP.

Accrediting organizations like The Joint Commission (TJC) and Leapfrog can further bolster appropriate glove use. TJC could incorporate glove stewardship into its metrics, such as adherence to indication-based glove use, HH compliance, and waste reduction. Leapfrog, whose focus is patient safety and quality, could incentivize institutions by linking proper glove use to accreditation and recognition programs. These bodies can catalyze widespread adoption of glove stewardship as a standard component of high-quality, climate-responsible care.

## Conclusion

The overuse of gloves represents a critical intersection of patient safety, IP, resource stewardship, and professional responsibility. Evidence shows that reducing inappropriate glove use not only improves HH but also lowers costs and decreases healthcare’s environmental footprint. Building on the successes of previous initiatives, healthcare organizations should adopt evidence-based guidelines and engage diverse stakeholders to develop glove campaigns tailored to their contexts. In doing so, we can safeguard patients, protect staff, and advance toward a more sustainable and ethical health system.

## Data Availability

No data was used for this article.
